# Finite element validation dataset of additively manufactured equal angle section stub columns

**DOI:** 10.1016/j.dib.2024.110318

**Published:** 2024-03-12

**Authors:** Ben Chater, Jie Wang, Mark Evernden

**Affiliations:** University of Bath, Claverton Down, Bath, BA2 7AY, United Kingdom

**Keywords:** Additive manufacturing, Finite element analysis, GMNIA, Local buckling, Stainless steel

## Abstract

This article provides experimental and numerical data pertaining to the compressive testing and model calibration for a novel design of 316 L stainless steel equal angle sections (EAS) produced through additive manufacturing, wherein each leg of the EAS is replaced by a wavy surface resembling high order buckling modes of the flat plate. The experimental data were acquired from testing 9 unique stub column sections, in all combinations of 3 different thicknesses and 2 wave magnitudes, with a control section provided for each thickness. The provided numerical data was produced to calibrate a finite element model of the tested sections by varying imperfection magnitudes, and selected values fit strongly to the physical tests. Both physical and numerical tests data herein are given in two parts each, one summary spreadsheet describing section geometry and peak load, and one more detailed spreadsheet providing load-displacement history for all physical sections and selected finite element sections. This data provides insight into finite element analysis of additively manufactured stainless steel sections, making it valuable for the validation of numerical models and stainless steel material behaviour.

Specifications Table

**Table:** 

Subject	Civil and Structural Engineering
Specific subject area	Geometric optimisation of non-prismatic steel structures.
Data format	Raw, Processed
Type of data	Spreadsheet
Data collection	The test data that is validated herein was gathered from an experiment undertaken in a compressive testing frame. Load data was collected from the frame itself, and displacements collected from LVDTs placed within the testing frame. A diagram within the article displays the test setup. One erroneous LVDT was excluded from the final data. Validation data was gathered from finite element simulations run in ABAQUS, with input files generated by a bespoke program.
Data source location	Institution: University of Bath Bath, UK. Physical tests were done at the University of Bath, and all numerical work was done using University computers and storage.
Data accessibility	Repository name: Figshare Data identification number: 10.6084/m9.figshare.24756876 Direct URL to data: https://figshare.com/s/74349361ac1f864f2d2e

## 1 Value of the Data

• These data demonstrate the performance of novel 3D printed stainless steel sections with regularly repeating non-prismatic geometry in compression at a variety of slendernesses.
• These data, despite the novelty of the sections they represent, may provide insight into the calibration of finite element models for stainless steel equal angle sections in compression.
• These data can be used by other researchers to calibrate their own SLM fabricated 316 L stainless steel models, avoiding the expense of fabricating their own samples before undertaking numerical studies.

## 2 Background

‘The dataset was compiled in order to calibrate GMNIA finite element models by replicating the physical specimen geometric and material properties and selecting suitable geometric imperfections for plated structures with wavy surface geometries. The current paper reports detailed experimental and numerical modelling process and methodology for other researchers to validate their numerical models when proposing various stiffening geometries. Studies on structural optimization of plated steel structures fabricated by selective laser melting may also employ the results of the current paper where physical tests are not possible. The proposed stiffening geometry also serves as an inspiration for structural stiffening in the construction industry, to delay or prevent local buckling when thin-walled design is the case, especially where digital manufacturing is employed.

## 3 Data Description

**Fig. 1:**
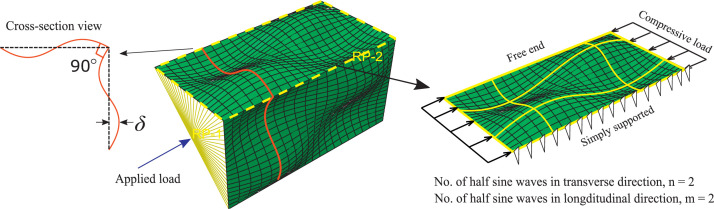
m2n2 EAS showing the notation of transverse and longitudinal half sine waves [1].

The presented data consists of 4 Excel spreadsheets pertaining to the physical compressive testing and numerical validation of 9 316 L stainless steel Equal Angle Section (EAS) stub columns, fabricated through Selective Laser Melting (SLM). 2 compressed files (.zip) containing the ABAQUS input files and geometric data point clouds of each stub column specimen have also been included in the repository. The selected stub columns use stiffening wave patterns consisting of 1 half-sine wave in the longitudinal direction, and 3 half-sine waves in the transverse direction, and are therefore considered to be m1n3 sections by the notation shown in Fig. 1. The contents of each spreadsheet are as follows:

1. **Physical_summary.xlsx -** Summary data of the 9 physical tests. These data include all relevant geometric parameters to fully describe the section, and the load and end displacement at failure. Geometric parameters include section length *L*, breadth *B,* thickness *t,* wave magnitude *δ*, longitudinal half-wave count *m*, and transverse half-wave count *n*.
2. **Physical_L-D.xlsx -** Load-displacement data for the 9 physical tests. These data include the full data produced by the data logger during the physical test, capturing load and end displacement at a polling rate of 1 Hz. This data has been lightly processed as described in the next section in order to eliminate various sources of inaccuracy, such as the stress response of the testing rig. The stress strain curves obtained from tensile coupon tests for each specimen thickness have also been included.
3. **Numerical_summary.xlsx -** Summary data of the numerical analysis. This includes load and end displacement for all tests used to calibrate the imperfection magnitude for the numerical models. Tested imperfection magnitudes were 0, B/1000, B/400, B/200, and B/100.
4. **Numerical_L-D.xlsx –** The material stress-strain curves used in the numerical models [2] and the Load-displacement data obtained from the calibrated numerical models. For the chosen imperfection values, where the numerical tests most closely resemble the physical tests, full load-displacement data are provided. This includes the load and displacement values for every single frame of the numerical analysis.
5. **Input_files.zip** – The ABAQUS input files used to generate the GMNIA finite element models for all the stub column specimens.
6. **Specimen_point_clouds.zip** – The scanned geometric data in the form of point clouds for all the stub column specimens.

## 4 Experiments, Numerical Simulations and Methodology

### 4.1 Physical experiments

A total of 9 equal angle section (EAS) samples with wave configuration m1n3 were fabricated using the Selective Laser Melting (SLM) method of additive manufacturing and tested in compression. The test set contains samples with length *L,* width *B*, 3 different section thicknesses *t* and two wave magnitudes *δ*, along with a prismatic control section for each thickness. Samples are referred to as EAS 50 × *T*-N; where T is 2, 3 or 4 and represents the sample's thickness, and N is F, 2.1 or 2.9 corresponding to the wave amplitudes; F representing flat for the control tests, and 2.1 and 2.9 representing amplitudes of 2.143 mm and 2.857 mm respectively.

The geometries of each stub column specimen were measured using both a vernier calliper and a digital scanner. The average measured properties are reported in **Physical_summary.xlsx.** The point clouds obtained from the scanning are given in **Specimen_point_clouds.zip** in the data repository.

**Fig. 2:**
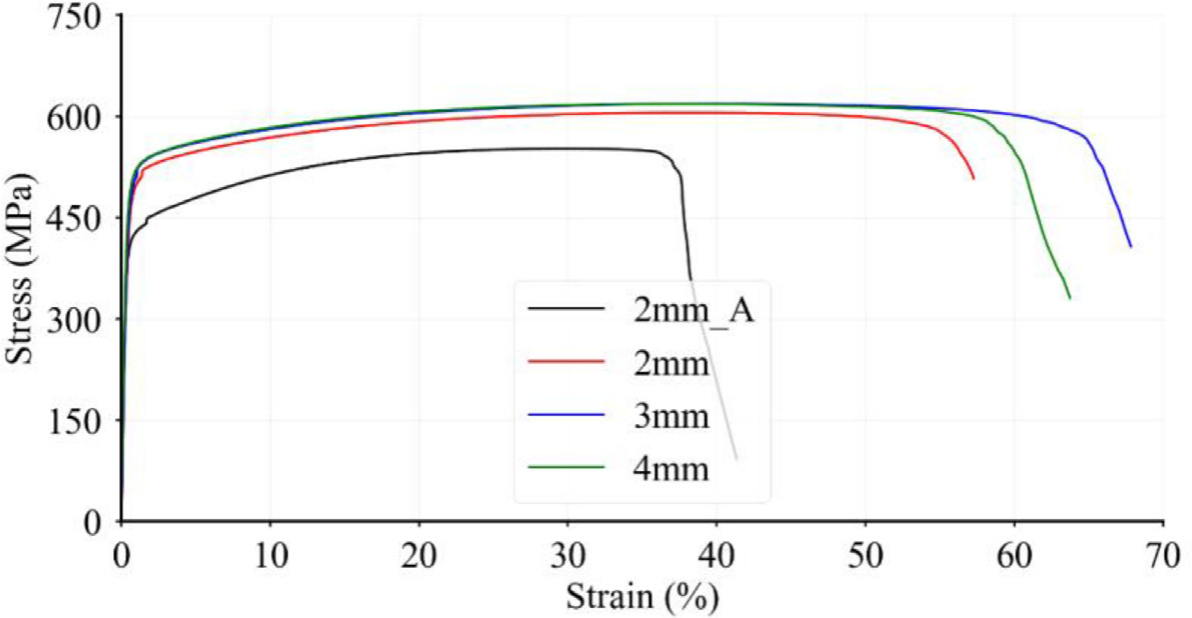
Stress-strain curves for 316 L tensile coupons, showing a single coupon from each batch.

Prior to stub column tests, tensile tests have been performed on coupons printed at the same time as the stub columns. The obtained stress-strain relationships are given in **Physical_L-D.xlsx**. It should be noted that 2mm_A and 2 mm in Fig. 2 represent two different batches of coupon specimens, where 2mm_A data is considered to be representative to chracterise the structural behaviour of 2 mm EAS stub columns.

**Fig. 3:**
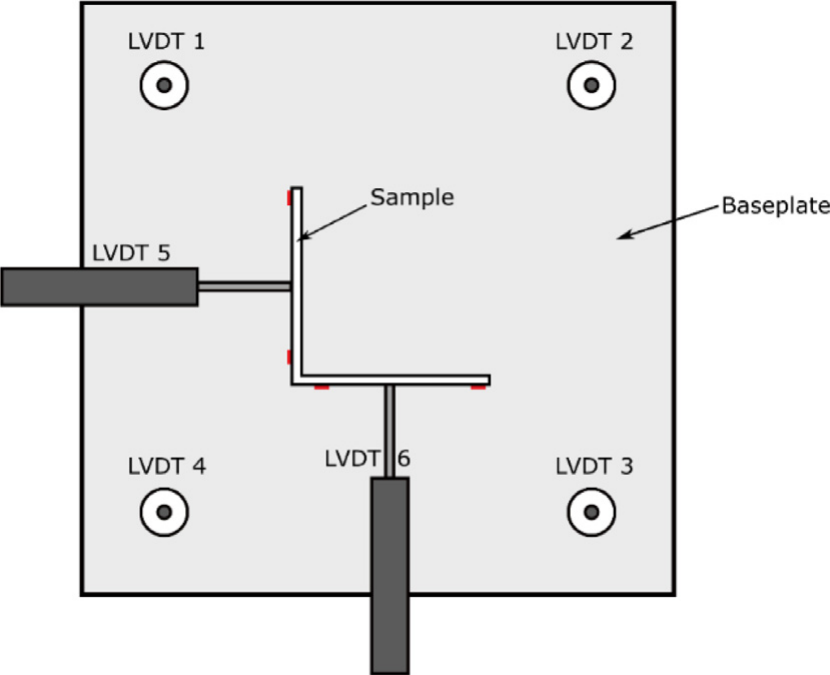
Plan view of stub column test setup. The red marks indicate locations of strain gauges (also at mid-height, vertically aligned) [3].

**Fig. 4:**
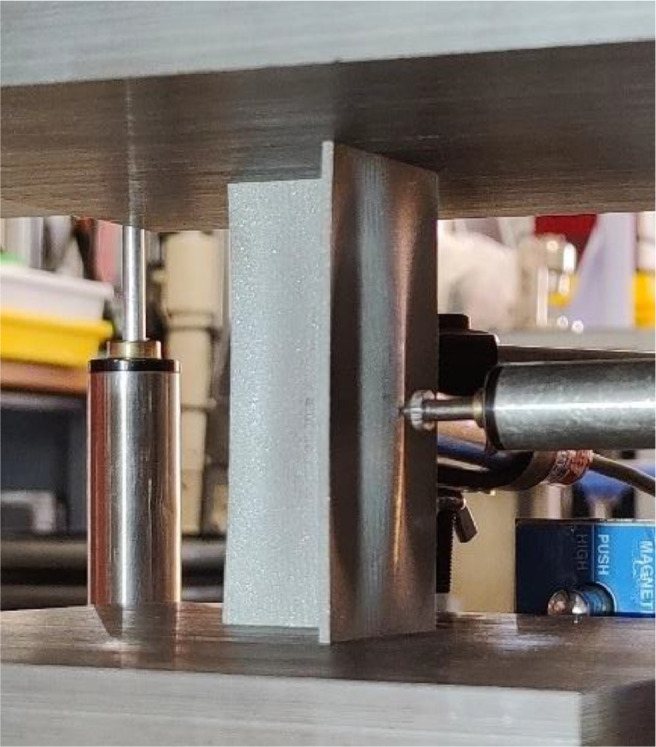
Image showing compressive testing of an m1n3 sample [3].

The compressive tests of the EAS stub columns were conducted in a Dartec 2000 kN testing frame. Measurements of applied load were provided by the testing frame, and 6 LVDTs were used to measure displacement with the arrangement shown in Fig. 3, Fig. 4. 4 LVDTs measured the vertical axial shortening and 2 LVDTs measured the horizontal displacement of the mid-point of each leg. The prismatic sections also carried 4 strain gauges arranged as in Fig. 3. These were omitted from the waved sections due to the curvature of the surfaces. Tests were displacement controlled, with a rate of 0.2 mm/min for the full duration and continued until the load reduced to below 90 % of the ultimate load achieved.

During processing, the LVDT displacement readings were compared against the strain gauge readings on the prismatic sections to produce stiffness values for the testing machine. Average strains were taken from the 4 gauges, and average displacement taken from the LVDTs. It is assumed that the strain measured by the gauges is representative of the average vertical strain across the full height of the section, and thus the shortening of the section can be calculated. This allows the machine-based displacement to be isolated from the effects such as the slight compression of the loading plates, columns, and other parts of the machine. Once machine stiffness has been calculated, it can be used for all tests including the non-prismatic sections to calculate strain on the section from just the LVDT readings. To ensure greatest accuracy, machine stiffness was calculated separately for each slenderness category. To produce a singular load-displacement plot for each test case, the average of the 4 vertical LVDT readings was used. LVDT 1 showed significant erroneous behaviour in almost every test case, and as such was omitted from the calculation for average displacement. The average sample displacement was then adjusted to account for the previously calculated machine stiffness. The load-displacement curves obtained from the tests are given in **Physical_L-D.xlsx**. The maximum load obtained in each test is given in **Physical_summary.xlsx**.

### 4.2 Numerical validation

The finite element models were produced and tested in ABAQUS [5]. Due to their non-standard geometry, the full generation process for the models is described herein. Models were generated via *.inp* input files (**Input_files.zip** in the data repository), which themselves were produced by a bespoke program. This program splits file generation into a number of steps:

(1) Node generation
(2) Element generation
(3) Node/element group generation
(4) Creating material/section properties
(5) End matter

Node generation was achieved by initially generating equivalent Node objects in the program (containing just coordinate data and an ID) in the positions required for a prismatic EAS. These nodes were evenly spaced, with distance and node count determined by a custom parameter, defining the number of nodes across the width of one leg of the EAS. Nodes were produced row-wise, starting each row on the tip of the x flange, and ending on the tip of the y flange.

After all nodes were generated, they were displaced out-of-plane by Eq. (1).

(1) $$\Delta \left(x,\;z\right)=\;\delta \cdot \sin\left(\frac{\pi nx}{B}\right)\cdot \sin\left(\frac{\pi mz}{L}\right)$$

Where:

x, z = local co-ordinates of an arbitrary point. z is distance along the section length; x is horizontal distance from the supported side.
∆(x, z) = local out-of-plane (y) displacement of a node at (x, z)
m, n = number of half sine waves in the length (longitudinal) and width (transverse) directions respectively.
L, B = section length and width respectively.
*δ* = maximum out-of-plane displacement; wave magnitude.

Elements are then generated connecting each node. S4R elements are used for these models, following standard convention for shell buckling tests [5]. Experimentally derived material data is then loaded into the program. Exact material properties chosen are dependent on the thickness of the material, as obtained from tensile coupon tests (as in **Physical_L-D.xlsx** and **Numerical_L-D.xlsx**). After material properties are loaded in, the rest of the input file is written, including step parameters, encastre boundary conditions on the stationary reference point (RP1), and restraint on all movement apart from the axial displacement of the moving reference point (RP2). A concentrated load is applied to RP2, and the outputs are defined including all default outputs and the initial volume for material use analysis.

**Fig. 5:**
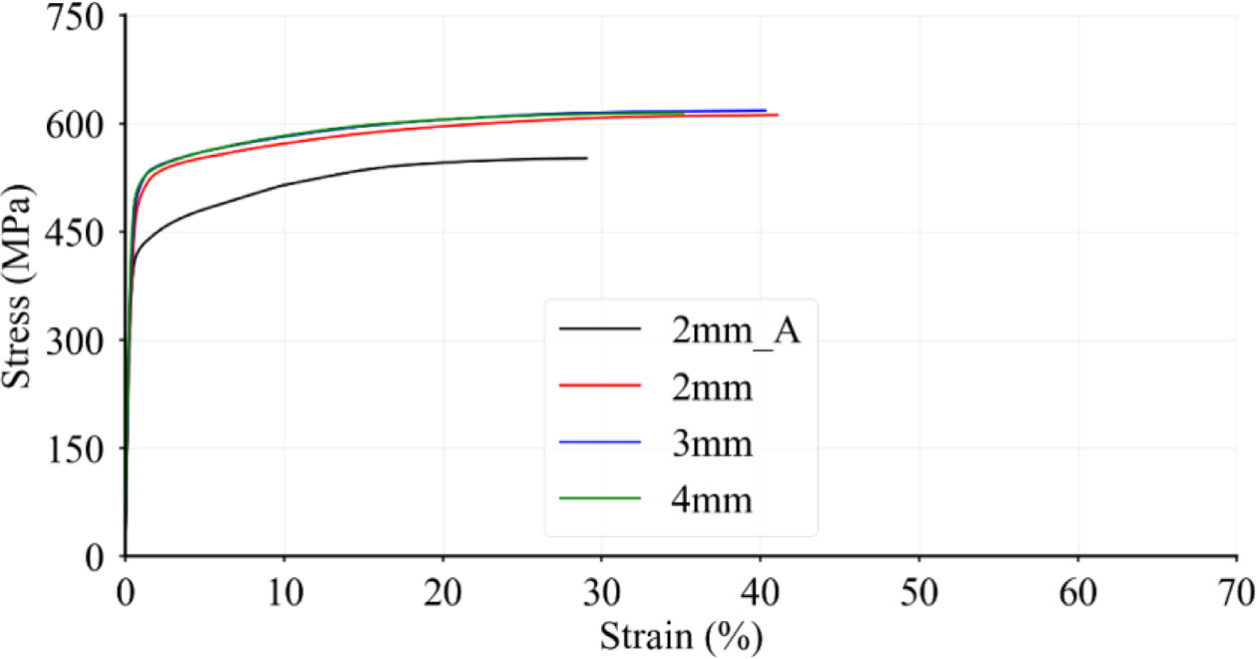
Constructed material curves, produced by fitting test data to the multi-stage hardening model described in [2].

The material properties used in the finite element models were reconstructed according to a modified R-O model described in [2,3]. This was because for each specimen thickness, three coupons were tested and to employ the average material properties, a reconstructed model must be used. The tabulated values of the reconstructed material model are given in **Numerical_L-D.xlsx** (Fig. 5).

Initial geometric imperfection was applied as the first eigen buckling mode of the plate (corresponding to the m1n1 shape as defined previously) with the magnitude calibrated for each section slendernesses. The imperfection was applied through super-positioning the wave pattern of m1n1 to the specimens. It should be noted that a various range of imperfection amplitudes have been explored, following the same logistic as in [6], and the amplitude offered the best match between the test and FE results was chosen for each section sizes. The magnitude used for the FE validation of each test is reported in **Numerical_summary.xlsx.**

**Fig. 6:**
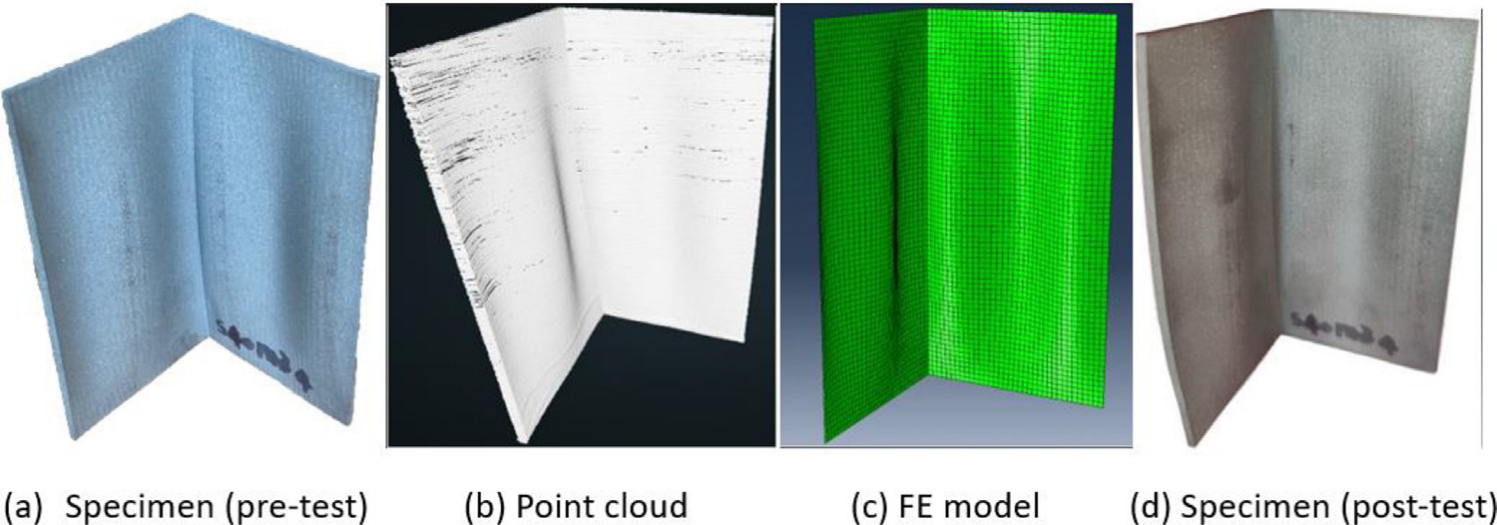
Comparison of test specimen, geometric scanned point cloud and FE model of specimen EAS 50 × 2–2.9.

The simulations were run using Static Riks method [4] and the data is collected by a Python script, which collects each individual test's data and records it into a text file for human interpretation and a JSON file for further processing. The specific outputs recorded are the initial volume, and for each frame of the loading step, the applied load and the displacement of RP2. The input files used to generate the models have been included in the data repository as **Input_files.zip**. These outputs have then been selected manually and arranged into the format described in the previous section. An example of comparison between the numerical model, the scanned geometry point cloud and the test specimen of EAS 50 × 2–2.9 are given in Fig. 6.

**Fig. 7:**
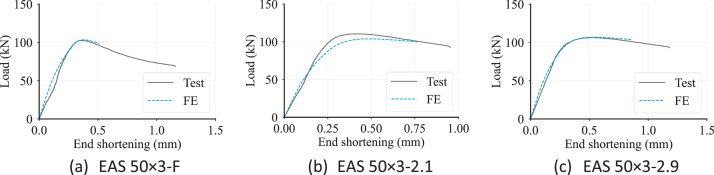
Load vs displacement curves obtained from the tests and FE models of typical specimens.

Typical comparisons between the load vs displacement curves obtained from the validated models and the corresponding test results are given in Fig. 7, indicating good agreement between the test and the FE models. More comparisons are given in the form of tabulated values in **Numerical_L-D.xlsx**.

## Limitations

The experimental and numerical results in the currently paper are only representative to the specific manufacturing process (i.e. selective laser melting) and the plate thicknesses (i.e. 2 mm, 3 mm and 4 mm) investigated, where a different manufacturing process such as wire arc additive manufacturing and different specimen thicknesses would produce different results, associated to the different material properties resulted.

## Ethics Statement

The authors have read and follow the ethical requirements for publication in Data in Brief and confirming that the current work does not involve human subjects, animal experiments, or any data collected from social media platforms.

## CRediT authorship contribution statement

**Ben Chater:** Conceptualization, Methodology, Software, Investigation, Formal analysis, Data curation, Writing – original draft. **Jie Wang:** Conceptualization, Validation, Resources, Writing – review & editing, Supervision, Project administration, Funding acquisition. **Mark Evernden:** Conceptualization, Resources, Writing – review & editing, Supervision.

## Data Availability

Finite element validation dataset of stub column compressive tests of slender SLM-fabricated 316L steel equal angle sections with non-prismatic geometry - Data in Brief (Original data) (Figshare)
